# The Nucleolus and PARP1 in Cancer Biology

**DOI:** 10.3390/cancers12071813

**Published:** 2020-07-06

**Authors:** Marina Engbrecht, Aswin Mangerich

**Affiliations:** Molecular Toxicology Group, Department of Biology, 78457 Konstanz, Germany; marina.engbrecht@uni-konstanz.de

**Keywords:** nucleolus, poly(ADP-ribosyl)ation, PARP, ARTDs, cancer

## Abstract

The nucleolus has been known for a long time to fulfill crucial functions in ribosome biogenesis, of which cancer cells can become addicted to in order to produce sufficient amounts of proteins for cell proliferation. Recently, the nucleolus has emerged as a central regulatory hub in many other cancer-relevant processes, including stress sensing, DNA damage response, cell cycle control, and proteostasis. This fostered the idea that nucleolar processes can be exploited in cancer therapy. Interestingly, a significant proportion of poly(ADP-ribose) polymerase 1 (PARP1) molecules are localized in the nucleolus and PARP1 also plays crucial roles in many processes that are important in cancer biology, including genome maintenance, replication, transcription, and chromatin remodeling. Furthermore, during the last years, PARP1 came into focus in oncology since it represents a promising target of pharmacological PARP inhibitors in various types of cancers. Here, we provide an overview of our current understanding on the role of PARP1 in nucleolar functions and discuss potential implications in cancer biology and therapy.

## 1. Introduction into the Biology of Nucleoli

Nucleoli are self-organizing, membrane-less sub-compartments of the nucleus, which are formed around tandemly repeated clusters of 200 to 400 ribosomal DNA (rDNA) genes known as nucleolar organizing centers (NORs) on the short arms of the five human acrocentric chromosomes 13, 14, 15, 21, and 22 [1,2]. In humans, nucleoli are surrounded by peri-nucleolar heterochromatin (PNH) derived from DNA sequences located distal and proximal to NORs [3]. The main function of nucleoli is ribosome biogenesis, which is one of the most energy-demanding and highly controlled processes in a cell. It has been estimated that in proliferating cells ribosome biogenesis consumes up to 80% of the cellular energy [4]. Therefore, it is not surprising that ribosome biogenesis is tightly coupled to the availability of growth factors, nutrients and cellular energy supply [5]. Nucleolar size positively correlates with rRNA synthesis and cell proliferation, as dividing cells often possess large nucleoli, while downregulation of rRNA gene transcription is associated with a reduction in nucleolar size [2]. Interestingly, an increased number and size of nucleoli have historically been used as a biomarker for tumor development [6].

Ribosome biogenesis starts with RNA polymerase I (Pol I)-driven synthesis of 47S pre-ribosomal RNA (pre-rRNA), which is rapidly processed by more than 200 non-ribosomal proteins and small nucleolar RNAs (snoRNAs) to mature 18S, 5.8S and 28S rRNAs [7]. These RNAs are assembled with ribosomal proteins (RPs) and 5S rRNA to form the pre-40S and pre-60S ribosomal subunits that are subsequently exported to the nucleoplasm to produce mature 80S ribosomes. Since the 5S rRNA is synthesized by RNA polymerase III (Pol III) and transcription of RP genes is mediated by RNA polymerase II (Pol II), ribosome biogenesis requires the activities of all three cellular RNA polymerases.

Light and electron microscopy have revealed the tripartite structure of the nucleolus, which reflects the different stages of ribosome biogenesis and is dependent on ongoing rDNA transcription [3,8]. During interphase, nucleoli consist of one or a few fibrillar centers (FC), of which each is surrounded by a dense fibrillar component (DFC) [9]. The FC contains non-transcribed rDNA sequences and transcription factors, e.g., the upstream binding factor (UBF), which recognizes the rRNA gene promotor. Transcription by Pol I occurs at the interface between FCs and the DFCs. DFCs are enriched in pre-rRNA processing factors, e.g., small nucleolar ribonucleoproteins (snoRNPs) and fibrillarin, and are the nucleolar sites, where early rRNA processing takes place. Both the FCs and the DFCs are embedded in a large granular component (GC), which is associated with late rRNA processing. Apart from this, mature rRNAs assemble in the GC with RPs into pre-ribosomal subunits [9].

Nucleoli are membrane-less biomolecular condensates thought to be organized by liquid-liquid phase separation [10,11]. This is supported by the finding that nucleoli of *Xenopus laevis* germinal vesicles fuse and turn over rapidly, thereby displaying liquid-like properties [12,13]. Mechanistically, molecular interactions between low-complexity, disordered protein regions and RNA molecules appear to contribute to such phase separation processes [14]. For a long time, an unresolved question has been, how the three nucleolar components can coexist without fusing to a single liquid phase. Recently, this issue has been addressed by Feric et al. [15] showing that nucleolar sub-compartments represent distinct liquid phases that can co-exist due to differences in the biophysical properties of their components, in particular due to distinct droplet surface tensions. Current evidence supports a model in which nucleoli are formed by a combination of active recruitment processes of proteins, active transcription of rDNA and formation of the respective rRNA, as well as phase separation processes involving both protein and RNA components [14].

In humans, nucleoli undergo structural reorganization throughout the cell cycle resulting in a transient loss of the tripartite structure. As cells enter mitosis, transcription of rDNA genes pauses, and nucleoli disperse. Interestingly, some nucleolar proteins, e.g., UBF, remain associated with NORs throughout the cell cycle, thus functioning as a mitotic bookmark and binding platform for the remaining Pol I transcription machinery [10,16]. Components of the DFC and the GC, such as fibrillarin and nucleophosmin, dissociate from the nucleolus during mitosis [17]. Before nucleoli reassemble in late anaphase or early telophase, these processing factors coalesce in foci designated as prenucleolar bodies (PNBs) [18]. The reassembly of nucleoli is initiated when pre-rRNA processing factors associate with NORs. First, multiple small nucleoli are formed, which as the cell cycle progresses coalesce to larger mature nucleoli [19].

Intriguingly, over the past two decades the nucleolus has emerged as a regulatory hub for multiple nuclear functions [20]. Over 4500 proteins were identified to be localized in nucleoli, of which only 30% are primarily associated with ribosome biogenesis [21,22]. This led to the assumption that the nucleolus has non-canonical regulatory functions beyond ribosome biogenesis. At present, it is well accepted that the nucleolus is a multifunctional nuclear sub-compartment, with additional roles, e.g., in stress response, DNA damage signaling, telomere maintenance, cell cycle control, cell proliferation, and proteostasis [23]. Furthermore, links between nucleolar functions and complex physiological and pathophysiological cellular processes have been established. For instance, there is increasing knowledge that the nucleolus is implicated in the aging process. In this context, several studies have postulated that rDNA instability could be a causative factor for aging [24]. Recently, the nucleolar size was shown to inversely correlate with longevity, thereby identifying small nucleoli as a potential, visible hallmark for longevity and metabolic health [4,25]. Moreover, there is growing evidence, that the nucleolus is involved in the development of neurodegenerative diseases, such as Parkinson’s disease [26]. Importantly, dysregulation of nucleolar functions has been linked to carcinogenesis, and consequently the nucleolus has emerged as a new target in cancer therapy [27,28,29,30]. This aspect will be discussed in more detail in Section 5 below. Before returning to this issue, in the next sections, we discuss how nucleoli respond to stress and DNA damage (Section 2), give a brief introduction into PARylation and PARP1 (Section 3), and review the role of PARP1 in nucleolar functions (Section 4).

## 2. How do Nucleoli Respond to Stress and DNA Damage?

Cells are constantly exposed to exogenous and endogenous sources of DNA damage [31]. To maintain genomic integrity, they possess a repertoire of repair proteins, which detect specific DNA lesions and initiate the appropriate repair pathway. Intriguingly, over 150 DNA repair proteins were identified in nucleoli under non-stress conditions, pointing to a role of nucleoli in DNA damage response [32].

As there is no structural barrier between the nucleolus and the surrounding nucleoplasm, proteins can, in principle, freely traffic from the nucleolus to the nucleoplasm and vice versa. The mechanisms by which proteins are retained in the nucleolus are still not fully understood. In general, it is assumed that nucleolar accumulation is a consequence of affinity interactions with RNA or nucleolar proteins. Some nucleolar proteins, e.g., nucleophosmin, exhibit a nucleolar localization sequence (NoLS) [33]. While there is no highly-conserved NoLS consensus sequence, about half of the amino acids (aa) inside NoLS are lysines and arginines, which render NoLS highly positively charged [23]. Since the nucleolus harbors high numbers of negatively charged RNA molecules, electrostatic interactions between NoLS and RNA molecules were postulated to be responsible for nucleolar accumulation of NoLS-bearing proteins. However, other nucleolar proteins lack a well-defined NoLS and are thought to be targeted to the nucleolus through protein-protein interactions with nucleolar anchored hub proteins, such as nucleophosmin or nucleolin [23].

The nucleolar accumulation of a variety of DNA repair proteins raised the question whether DNA repair proteins are merely sequestered in the nucleolus for storage reasons until they are required for their functional role in DNA repair, or if they also fulfill nucleolar-specific functions. Indeed, at least for some DNA repair proteins, including the base excision repair (BER) protein apurinic/apyrimidinic endonuclease 1 (APE1) and the RecQ helicase WRN, nucleolar-specific functions were postulated [32,34]. APE1 was not only shown to reside in the nucleolus and interact with nucleophosmin, there is also growing evidence that it is involved in quality control of the transcribed rRNA [35]. The gene encoding for the Werner protein (WRN) harbors mutations in Werner syndrome, an adult onset progeria characterized by premature aging and cancer development [36]. WRN is involved in a number of DNA repair pathways, including homologous recombination (HR), non-homologous end joining (NHEJ) and BER [37,38]. Under non-stress conditions, it localizes to transcriptionally active nucleoli, giving rise to the idea that WRN might also play a role in ribosome biogenesis [39]. Further studies supported this notion in demonstrating that WRN co-immunoprecipitates with the RNA Pol I subunit RPA40 and that in the absence of WRN, 18S and 28S RNA levels are reduced [40]. Although the findings require further elucidations, these data provide convincing evidence that bona fide DNA repair proteins might play dual roles in DNA repair on the one hand and ribosome biogenesis on the other hand.

Nucleoli are highly dynamic and undergo dramatic structural changes upon suffering various types of DNA damage. A plethora of cellular stressors, including UV and γ radiation, oxidative stress, genotoxic chemotherapeutic agents, hypoxia, as well as nutrient and growth factor deprivation can induce nucleolar re-organization or disruption [41]. Any stress-induced perturbation in ribosome biogenesis that ultimately leads to disruptions in cell homeostasis through activation of p53 or other stress signaling is referred to as nucleolar—or ribosomal stress [42]. Induction of nucleolar stress can occur at various steps of ribosome biogenesis, from Pol I transcription to pre-RNA processing, and eventually to assembly of ribosomal subunits and their release from the GC [42]. For instance, treatment with actinomycin D (ActD), which at low doses acts as a selective inhibitor of RNA Pol I by intercalating with G/C-rich rDNA, was shown to induce nucleolar segregation and the formation of so called nucleolar caps [14]. Nucleolar caps are bipartite structures that are excluded from the GC and reside at the nucleolar surface. They consist of FC and DFC components, with the DFC facing the nucleolar interior and the FC facing the nucleoplasm [3]. Interestingly, a similar reorganization of nucleolar structure was observed upon targeted induction of DSBs in rDNA repeats, which was shown to result in ataxia telangiectasia mutated (ATM)-dependent inhibition of RNA Pol I transcription [43,44,45]. Other studies reported that rDNA DSB-induced Pol I inhibition and nucleolar cap formation can also be regulated by the DNA-dependent protein kinase (DNA-PK) [46,47]. The formation of nucleolar caps was proposed to render damaged rDNA accessible for repair factors that are normally excluded from nucleoli under non-stress conditions [48]. In three recent studies, the *Physarum polycephalum* homing endonuclease I-*Ppo*l, which can cleave within the 28S rRNA coding region in human cells, was used to investigate whether rDNA DSBs are repaired by HR or NHEJ [49]. Harding et al. identified NHEJ as the predominant pathway in the repair of DSBs within rDNA, since depletion of NHEJ, but not HR factors, leads to accumulation of DSBs in the nucleolus [44]. Instead, van Sluis et al. [45] demonstrated that HR is also involved in rDNA DSB repair. Interestingly, HR factors are recruited to stress-induced nucleolar caps even in G1 cells, suggesting that in rDNA, HR occurs even in the absence of sister chromatids, possibly by using other rDNA repeats as a template. Therefore, the formation of nucleolar caps could facilitate repair of rDNA via HR by concentrating high levels of homologous sequences in close proximity [50]. Interestingly and in contrast to the general notion that HR acts as an error-free repair mechanism, it was reported that the HR-mediated repair of rDNA is error-prone, leading to a reduction of rDNA repeats, thus promoting rDNA instability [51].

Ribosomal DNA sequences are especially vulnerable to DNA damage due to their repetitive nature, the unique organization of rDNA in clusters on five different chromosomes and high transcription rates [17]. These special features render the rDNA prone to unscheduled DNA recombination events and frequent formation of DNA:RNA hybrids, which are thought to favor the generation of DSBs [52,53]. If not properly repaired, rDNA damage can give rise to rDNA instability, and therefore contribute to premature onset of disease and carcinogenesis. Indeed, 50% of lung and colorectal cancers were shown to harbor rDNA gene rearrangements [54]. The pathways involved in rDNA damage repair and their regulatory mechanisms still require further investigations to gain a deeper insight into the mechanisms, which drive rDNA instability and ultimately carcinogenesis.

Perturbations in nucleolar functions are often accompanied by the release of nucleolar and ribosomal proteins into the nucleoplasm, where they are proposed to take on secondary functions in stress response. For instance, nucleophosmin was shown to shuttle between the nucleolus, the nucleoplasm, and the cytoplasm upon induction of stress [55,56]. Nucleolar release of nucleophosmin can be induced by a variety of DNA-damaging agents, including ionizing radiation (IR), cisplatin or etoposide treatment [23]. Interestingly, it was postulated that nucleophosmin takes over moonlighting functions in the nucleoplasm by participating in several DNA repair pathways, including BER and nucleotide excision repair (NER) [57]. Additionally, nucleophosmin drives p53 signaling upon genotoxic stress. Nucleophosmin constitutively interacts with the key activator of the p53 signaling pathway ARF, thus targeting it to the nucleolus [58]. Upon genotoxic stress, modification of nucleophosmin leads to the release of ARF into the nucleoplasm, where it inhibits HDM2, the E3 ligase that negatively regulates p53 [17]. Additionally, nucleophosmin regulates the HDM2-p53 pathway in an ARF-independent manner, by direct protein-protein interactions with HDM2 [59]. It is important to note that while most proteins are released from nucleoli under stress conditions, there are some notable exceptions. For example, upon stress, the promyelocytic leukemia tumor suppressor (PML) protein sequesters Mdm2 to the nucleolus by nucleolar translocation, thereby stabilizing p53 in the nucleoplasm, and promoting apoptosis [60]. Furthermore, stress-induced ubiquitination of the NF-κB subcomponent RelA leads to its translocation to the nucleolus, by which this process elicits pro-apoptotic effects [61]. Interestingly, also Hsp70 and other chaperone proteins accumulate in the nucleolus during stress conditions, suggesting to protect nucleolar proteins from aggregation [62,63,64,65]. In this regard, a recent study by Frottin et al. [63] addressed the role of liquid-liquid demixing and phase transition processes during nucleolar stress response. The authors reported that the liquid-like GC phase of the nucleolus can act as a quality control compartment by exerting transient chaperone-like activity for nuclear proteins entering the nucleolus during stress conditions [63].

Apart from proteins that have predominantly nucleolar functions, several DNA repair factors, including APE1 and WRN, have been shown to undergo DNA damage-induced nucleolar-nucleoplasmic shuttling [23,40,66]. Despite extensive research the exact molecular mechanisms, by which proteins are released from the nucleolus remain poorly understood. In general, it is assumed that such protein dynamics are regulated by a network of PTMs and protein-protein interactions, which is likely to occur in a stress- and protein-specific manner. For instance, Karmakar et al. [67] showed that WRN is acetylated upon treatment with mitomycin C and the alkylating agent methyl methane-sulfonate (MMS), but not after UV irradiation. In addition, phosphorylation was proposed to modulate WRN’s subnuclear localization [39,68]. Consistent with the idea that such DNA damage-induced protein dynamics are regulated in a stress-specific manner, we recently found that after treatment with H_2_O_2_ and the alkylating agent 2-chloroethyl ethyl sulfide (CEES), but not the topoisomerase inhibitor camptothecin (CPT), nucleolar-nucleoplasmic translocation of WRN was dependent on the PARP1 protein, yet independent of its enzymatic activity [69].

## 3. Introduction into the Biology of PARP1 and Poly(ADP-ribosyl)ation

Post-translational modifications (PTMs), such as phosphorylation, acetylation or ubiquitination, represent an extremely versatile and fast means of regulating the complexity and dynamics of cellular processes. In particular, ADP-ribosylation, which can be found in most eukaryotes and which has been shown to be essential during mammalian development [70], represents a highly dynamic and fully reversible PTM [70,71,72,73]. This modification is catalyzed by ADP-ribosyl transferases to which the family of poly(ADP-ribose) polymerases (PARPs) belongs. The PARP gene family comprises 17 members in humans and based on structural homology of their catalytic domain with the diphteria toxin, they are also referred to as ADP-ribosyltransferases diptheria toxin-like (ARTDs) [74]. By using nicotinamide adenine dinucleotide (NAD^+^) as a substrate, PARPs covalently attach ADP-ribose units to a variety of aa residues of acceptor proteins, including serines, glutamates, aspartates, lysines, and tyrosines [75,76,77,78,79,80,81,82]. While most PARPs possess mono(ADP-ribosyl)transferase activity (mono(ADP-ribosyl)ation, MARylation) or are catalytically inactive, at least four of them, i.e., PARPs 1, 2, 5a, 5b, are known to synthesize negatively charged polymers of poly(ADP-ribose) (PAR), which earned them the term ‘writers’ of poly(ADP-ribosyl)ation (PARylation)—(PARPs 5a and 5b are also known as tankyrases 1 and 2). The resulting PAR chains are either linear or branched and may consist of more than 200 ADP-ribose moieties [83,84,85]. Apart from covalent modification, certain proteins—so called ‘readers’ of PARylation—can non-covalently interact with PAR chains via distinct PAR binding modules, which, among others, include loosely conserved PAR binding motifs (PBM), zinc finger-type structures, macrodomains, WWE domains and OB folds [86].

PARP1—the founding member of the PARP family—was extensively studied since the discovery of PARylation in 1963 [87]. It plays pivotal roles in various cellular processes, including chromatin remodeling, replication, transcription, RNA biology, energy metabolism, immunity and inflammation, as well as cell death [88,89,90,91,92]. In addition, one of the most critical functions of PARP1, is its role in DNA repair and genome maintenance. Thus, PARP1 participates in several DNA repair pathways, including BER, NER, as well as the two double-strand break (DSB) repair pathways, i.e., NHEJ and HR [88,93,94]. Furthermore, PARP1 was shown to recognize unligated Okazaki fragments and to stabilize stalled replication forks, owing to promote repair during replication and replicative stress [95,96]. The importance of PARP1 in DNA repair is impressively exemplified by the fact that *Parp1*-deficient mice are hypersensitive towards DNA damaging agents, which is accompanied by increased spontaneous as well as induced genomic instability and carcinogenesis [97]. On a molecular level, PARP1 acts as a sensor of DNA damage, in particular DNA single and double strand breaks, and detects those via certain zinc finger motifs of its N-terminal DNA binding domain. Binding to DNA strand breaks induces allosteric conformational changes in the protein structure, which allow binding of NAD^+^ to the C-terminal catalytic domain, leading to the enzymatic activation of PARP1 [71,85,98]. Indeed, PARP1 was reported to be responsible for over 90% of DNA damage-induced PARylation, whereupon cellular PAR levels dramatically increase 10- to 500-fold compared to the PAR levels under non-stress conditions [88,99,100]. Apart from DNA damage-dependent activation, PARP1 activity is regulated by other PTMs, such as acetylation, phosphorylation and SUMOylation [101,102,103,104] as well as physical protein-protein interactions [85]. The first and main target of PARP1-dependent PARylation is PARP1 itself [105,106]. Such automodification of PARP1 provides a platform for the recruitment of downstream factors, i.e., ‘readers’ of PARylation, of which the DNA repair protein XRCC1 represents a prime example [107,108,109]. There is now considerable evidence that high-affinity, non-covalent interaction of proteins with auto-PARylated PARP1 mediates substrate specificity of PARP1 and targets these PAR-binding proteins to subsequent covalent modification by PARP1 [110,111,112]. In total, several hundred PARylated proteins have been identified, which are involved in diverse cellular functions, ranging from genome maintenance, DNA damage response and chromatin organization to transcription, RNA metabolism and cell cycle regulation [80,82,113].

Eventually PARP1 is released from DNA, which is assumed to occur due to steric and electrostatic repulsion of the automodified protein, yet other, more specific and so far largely unexplored, mechanisms may be conceivable as well [88,114,115]. Importantly, in many instances and in particular upon genotoxic stress, PARylation is transient, highly dynamic, and fully reversible, since after being synthesized, PAR chains are rapidly degraded in a two-step process, due to the enzymatic activities of certain ‘eraser’ enzymes [116,117]. Thus, the bulk of DNA damage-induced PAR can be degraded by poly(ADP-ribose) glycohydrolase (PARG), which harbors exo- as well as endo-glycosidic activities for PAR. The most proximal, protein-bound ADP-ribose moiety, however, cannot be released by PARG. This is instead carried out by several other eraser enzymes, which possess individual specificities for certain ADP-ribose acceptor sites, e.g., ARH3 can remove ADP-ribose from serine, and MacroD1/MacroD2 from glutamate or aspartate [116,117,118,119,120,121]. Moreover, the terminal ADP-ribose protein glycohydrolase 1 (TARG1/C6orf130) was shown to act on glutamate and aspartate residues, as well, by removing and releasing not only ADP-ribose, but also entire PAR chains [116,122].

In general, PARylation is thought to regulate the physicochemical properties, localization, and enzymatic activities of its target proteins in a highly controlled, spatio-temporal manner. Furthermore, PAR can serve as a signal to target proteins for degradation through the ubiquitin-proteasome system [123], which can, therefore, act as a ‘kiss of death’ for certain factors during DNA damage response. Another layer of complexity in the functions of PARylation is given by findings demonstrating that PAR can act as a seed for liquid-liquid demixing processes, thereby triggering the formation of biomolecular condensates [124,125]. In this regard, it has also been reported that such processes contribute to the formation of dynamic protein foci at sites of DNA damage, thereby facilitating DNA repair processes through transient and functional compartmentalization of DNA damage sites [110,124]. Moreover, there is mounting evidence that PARylation and PAR-binding regulate liquid-liquid phase separation and aggregation of several neurodegenerative disease-associated RNA-binding proteins, including α-synuclein, TDP-43 and hnRNP A1 [126]. In summary, it is assumed that PARP1 orchestrates and supports DNA damage response mechanisms and local chromatin dynamics. In addition, beyond its role in the control of protein localization and biochemistry, PARP1 is involved in the regulation of cell death and cellular energy metabolism, i.e., by using NAD^+^ as a substrate, for which reason it proves itself as a global regulator of cellular physiology and pathophysiology [92,127].

Over the past decades, PARP1 came into focus as a target in clinical oncology, since PARP inhibitors were identified to act as chemosensitizers in combination with classical DNA-damaging therapies or as monotherapeutic agents to treat cancers with defects in HR repair according to the concept of synthetic lethality. In 2005, two independent groups discovered the synthetic lethal interaction between PARP1 inhibition and loss of BRCA, i.e., *BRCA1* or *BRCA2* [128,129], which spurred the development of clinical PARP inhibitors. By now, four of such small-molecule PARP inhibitors (i.e., olaparib, rucaparib, niraparib, and talazoparib) have been approved by authorities such as the EMA and the FDA to treat certain types of ovarian, breast or pancreatic cancer with germline loss-of-function mutations of *BRCA* genes [130,131]. Functional BRCA1 and BRCA2 are of critical importance for the repair of DSBs via HR [132]. Inhibition of PARP not only leads to accumulation of DNA single-strand breaks (SSBs), but also to trapping of PARP at the DNA, which may result in toxic manifestations of the damage and stalled replication forks [133]. Due to PARP inhibition and the absence of BRCA, the stalled replication forks cannot be restarted properly, leading to replication fork collapse and ultimately DSBs, which are lethal for HR-deficient cancer cells [134]. By selectively targeting certain types of cancers, PARP1 inhibitors provide a successful step towards precision medicine in oncology. In addition to their use in *BRCA*-mutated cancers, there is meanwhile good evidence for further synthetic lethal interactions of PARP inhibitors in combination with other genetic constellations. Moreover, the use of PARP inhibitors as chemosensitizers represents a promising strategy for their use in cancer treatment [130,131,132]. Taken together, it is expected that the area of application for the use of PARP inhibitors will further expand during the next decade and that PARP inhibitors will find their place in the range of chemotherapy regimens.

## 4. On the Role of PARP1 and PARylation in the Biology of the Nucleolus

Under non-stress conditions a substantial percentage of cellular PARP1 molecules (i.e., ~40%) reside within nucleoli [135]. Nucleolar accumulation of PARP1 was firstly documented in the late 1980s by using immunolabeling and was later on confirmed in proteomic studies [136,137]. PARP1, as well as PARP2, are retained in nucleoli via interaction with the multifunctional nucleolar hub protein nucleophosmin, which is implicated in multiple steps of ribosome biogenesis, including rDNA transcription and elongation, as well as rRNA processing [32,138]. Treatment with the RNA Pol I inhibitor ActD resulted in nucleolar release of PARP1 and PARP2, indicating that active nucleolar transcription is required for PARP1 and PARP2 to reside in nucleoli [139]. The presence of PARP1, particularly in transcriptionally active nucleoli, gave rise to the idea that PARP1 might be involved in canonical nucleolar functions, e.g., in regulating ribosomal biogenesis. Indeed, there is a growing body of evidence, that PARP1 and PARylation play important roles in nucleolar biology, which will be discussed in the following.

**Figure 1:**
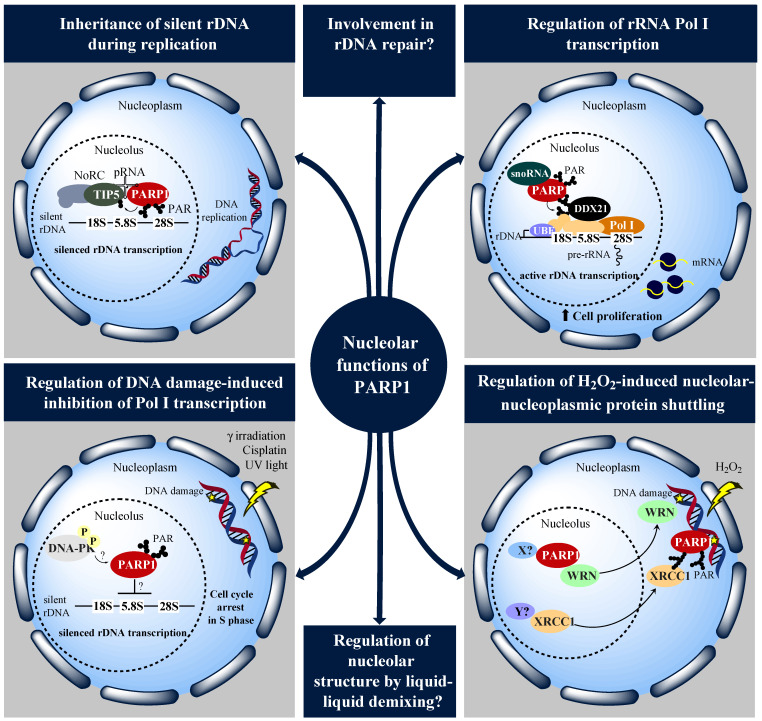
Functional roles of PARP1 in nucleolar biology. DDX21, DEAD-box helicase 21; DNA-PK, DNA-dependent protein kinase; NoRC, nucleolar remodeling complex; PAR, poly(ADP-ribose); PARP1, poly(ADP-ribosyl) polymerase 1; Pol I, polymerase I; pRNA, promotor-associated RNA; rDNA, ribosomal DNA; rRNA, ribosomal RNA; snoRNA, small nucleolar RNA; TIP5, TTF-1-interacting protein 5; UBF, upstream binding factor; WRN, Werner syndrome protein; XRCC1, X-ray repair cross complementing 1. For details see Section 4.

First evidence for PARP1 to play a role in nucleolar biology was proposed by Tulin et al. [140] in 2002 by using *Drosophila melanogaster* as a model system. Unlike mammals, the *Drosophila* genome contains only two PARP encoding genes, i.e., one that is highly related to mammalian PARP1, as well as one homolog of tankyrases [141,142]. Therefore, *Drosophila* is a powerful model organism to study PARP biology. In the study of Tulin et al. [140], it was shown that many of the PARylated proteins in *Drosophila* are enriched in nucleoli and in the heterochromatic chromocenter regions. Furthermore, disruption of PARP1 expression resulted in abrogated formation of nucleoli and larval lethality, suggesting that PARP1 is required for the formation of nucleoli during development [140]. As previously discussed, it was shown that liquid-liquid phase separation plays an important role in internal organization of nucleolar architecture [3]. Interestingly, PAR can function as a seed for liquid-liquid demixing in intrinsically disordered proteins, which are abundant in nucleoli [125]. Therefore, it is conceivable, that PARylation regulates the general biophysical state of nucleolar architecture by liquid-liquid demixing [124] (Figure 1). In this regard it is interesting to note that nucleoli in yeast and other lower eukaryotes exhibit a bipartite structure, i.e., lacking the FC in nucleoli [143]. The fact that PARylation is missing in yeast [144] makes this an interesting correlation, which needs to be analyzed in detail for any causative relationship in future studies.

Meanwhile, several studies have revealed an important role of PARP1 in regulating multiple steps of ribosome biogenesis. Thus, PARP1 was proposed to participate in ribosome biogenesis by controlling pre-rRNA processing, post-transcriptional modification, and assembly of pre-ribosomal subunits [145]. Disruptions of PARP1 enzymatic activity led to nucleolar disintegration and aberrant localization of nucleolar-specific proteins, such as fibrillarin, nucleophosmin and nucleolin. Therefore, the authors of this study concluded that PARP1 and PARylation are important for nucleolar integrity and the localization of nucleolar-specific proteins in proximity to pre-rRNA. Furthermore, PARP1 mutants displayed a delay in rRNA processing and increased levels of rRNA intermediates, such as 47S and 36S, resulting in decreased ribosome levels [145]. In mammalian cells, it was demonstrated that PARP1 participates in nucleolar remodeling complex (NoRC)-mediated rDNA silencing during replication [146] (Figure 1).

In mid-late S phase, the NoRC mediates heterochromatin formation and silencing of rDNA transcription via the recruitment of the histone acetylase HDAC1 and DNA methyltransferase (DNMT) to the rDNA promoter [147]. PARP1 associates with the rDNA repressor TTF-1-interacting protein 5 (TIP5), which is part of NoRC, at silent rRNA genes during replication [146].

Furthermore, association of PARP1 with TIP5 is mediated by the NoRC-associated noncoding RNA (pRNA, promoter-associated RNA). Interestingly, PARP1 PARylates silent chromatin and components of the NoRC complex, including TIP5. Thus, it is likely that pRNA stimulates PARP1 enzymatic activity, which is necessary to establish rDNA silencing. These findings indicate that PARP1 can modulate chromatin structure and gene expression in nucleoli, revealing a mechanism by which PARP1 ensures that silent rDNA regions are properly inherited after their disruption during DNA replication [148,149]. In a recent study, another pathway was identified by which nucleolar actions of PARP1 participate in the control of rDNA transcription and ribosome biogenesis during cell proliferation [112]. Thus, Kim et al. [112] demonstrated that PARP1’s binding to snoRNAs leads to its catalytic activation in a DNA damage-independent manner. Upon activation of its catalytic activity, PARP1 ADP-ribosylates the nucleolar RNA helicase DDX21, which results in enhanced rDNA transcription and proliferation of breast cancer cells (Figure 1). As discussed below in more detail, the pharmacological inhibition of this pathway may contribute to the effectiveness of PARP inhibitors in the treatment of certain cancers [112].

Importantly, there is emerging evidence that PARP1 and its enzymatic activity are involved in the regulation of ribosome biogenesis under certain pathological conditions. For instance, in hippocampal pyramidal neurons in Alzheimer’s disease (AD) nucleolar PARP1 is significantly decreased compared to control cells [150]. It was proposed that under physiological conditions, PARP1 ADP-ribosylates DNMT1, thereby preventing rDNA methylation, which results in upregulation of rDNA transcription. Thus, in AD neurons, PARP1 mislocalization leads to hypermethylation of rDNA, reduced rDNA transcription and impaired ribosome biogenesis, which ultimately results in disruption of long-term memory formation. Interestingly, such a decrease in PARP1 staining was revealed in neurons of individuals with mild cognitive impairment (MCI), suggesting that decreased nucleolar PARP1 could act as an early biomarker of cognitive impairment [151]. For a more detailed discussion on this topic and the role of PARPs and PARylation in RNA biology in general the reader is referred to a comprehensive recent review by Kim et al. [91].

Apart from its contribution to ribosome biogenesis under non-stress conditions, PARP1 was shown to participate in the nucleolar stress response, in particular DNA damage response. A study by Calkins et al. [152] demonstrated that PARP1 regulates rDNA transcription in response to DNA damage (Figure 1). Induction of DNA damage, by using γ irradiation, UV light or the cross-linking agent cisplatin, resulted in inhibition of rDNA transcription, as well as cell cycle arrest in S phase. Inhibition of PARP1 or DNA-PK prevented silencing of rRNA synthesis, yet not the accumulation of cells in S phase. These results indicate that PARP1 and DNA-PK are involved in DNA damage-induced inhibition of rDNA transcription, however not in the accompanying cell cycle arrest. Loss of DNA-PK function prevented PARP1 from being activated and recruited to chromatin, suggesting that DNA-PK acts upstream of PARP1 to block rRNA synthesis upon DNA damage. While the exact mechanistic details by which PARP1 and DNA-PK contribute to DNA damage-induced inhibition of rDNA transcription remain to be elucidated, PARP1 may also facilitate DNA damage-induced block of rRNA synthesis through the recruitment of nucleolar proteins with roles in ribosome biogenesis to the nucleoplasm. For instance, fluorescence loss in photobleaching (FLIP) experiments showed that the macrodomain-containing protein TARG1/C6orf130 continuously undergoes nucleolar-nucleoplasmic shuttling [153]. Interestingly, the distribution of TARG1/C6orf130 between these two compartments was regulated by PARylation. Thus, in the absence of PAR, TARG1 localizes to transcriptionally active nucleoli, while in response to DNA damage-induced PAR formation, e.g., upon H_2_O_2_ treatment, it re-localizes to the nucleoplasm. Since TARG1 was reported to bind to RNA, ribosomal proteins, as well as proteins associated with rRNA proteins and ribosomal assembly factors, it is conceivable that in nucleoli, TARG1 plays a role in ribosome assembly or quality control, which is stalled when TARG1 is recruited to sites of DNA damage in a PAR-dependent manner [153]. With regards to a role of PARP1 and PARylation in nucleolar DNA damage response, we recently showed that upon H_2_O_2_ treatment of HeLa cells, WRN and XRCC1 translocate from nucleoli to the nucleoplasm, however interestingly enough, probably by different mechanisms [69]. Thus, while the release of WRN from nucleoli was purely dependent on the presence of PARP1 protein without any obvious involvement of its catalytic activity, we found that relocalization of XRCC1 upon H_2_O_2_-induced DNA damage was dependent on both PARP1 protein and its enzymatic activity (Figure 1). We hypothesized that in case of WRN, PARP1 and an additional unknown factor mediate the release of WRN from nucleoli, while XRCC1 requires nucleoplasmic DNA damage-bound and PARylated PARP1 as a loading platform, which leads to XRCC1 retention in the nucleoplasm until its tasks in BER are completed. This notion is supported by findings revealing that in cells without PARP1 activity, XRCC1 relocates quickly to nucleoli upon DNA damage induction [69].

Similarly to XRCC1, upon induction of DNA damage the ribosomal protein L6 (RPL6) is recruited to sites of DNA damage in a PARP-dependent manner [154]. At sites of DNA damage RPL6 appears to regulate the DNA damage response. Thus, RPL6 directly interacts with histone H2A and depletion of RPL6 impairs the recruitment of the mediator of DNA damage checkpoint 1 (MDC1), reduces ubiquitination of H2A and phosphorylated histone H2AX (γH2AX). These results exemplify that ribosomal proteins can exert PARP-dependent extraribosomal functions in DNA damage response. In general, these results support the notion that nucleolar-nucleoplasmic shuttling mechanisms are mediated by several different processes, which are highly dependent on the specific stress condition as well as the specific protein.

Another layer of complexity is added by a study of Leger et al. [155], which showed that H_2_O_2_ and N-methyl-N’-nitro-N-nitrosoguanidine (MNNG) induce PAR formation in nucleoli. Interestingly, the combination of H_2_O_2_ or MNNG treatment with low doses of ActD revealed a synergistic effect on nucleolar PAR formation. At low concentrations ActD intercalates with GC-rich sequences of rDNA downstream of rDNA transcription start sites. Thus, ActD prevents transcription at the elongation step, resulting in accumulation of short rRNA transcripts. Interestingly, this study reported that PARP2, but not PARP1, binds through its N-terminal SAP domain to these short rRNA transcripts and thereby becomes activated, which contributes to the enhanced PAR formation inside nucleoli [155].

## 5. Implications for Cancer Biology

The first link between nucleoli and cancer was established over a century ago, when pathologists noticed that nucleoli in cancer cells are often enlarged, irregularly shaped, as well as increased in number and therefore could serve as markers of aggressive malignancies [156]. In general, the roles of nucleolar processes in cancer biology are manifold and for a more in-depth discussion on this topic the reader is referred to comprehensive recent review articles [6,17,29,30,157]. Broadly speaking, the role of nucleoli in carcinogenesis falls into two interdependent categories: ribosome biogenesis and stress response. The current consensus is that structural abnormalities of nucleoli in cancer cells are the direct consequence of increased ribosome biogenesis, which goes along with aberrant Pol I transcription [157]. Thus, in a way, tumor cells depend on increased ribosome biogenesis to reach their demand for newly synthesized proteins during cell proliferation [6]. Interestingly, rDNA gene clusters represent hot spots of recombination in human cancer [6]. Thus, more than half of solid tumors such as lung or colorectal cancer exhibit rDNA rearrangements [54] and alterations are also frequently observed in Hodgkin’s lymphoma [158].

Usually cells precisely monitor the accuracy of ribosome biogenesis, as well as nucleolar integrity, and disruption at any step of ribosome biogenesis results in activation of cellular checkpoints [27]. Thus, while the increased ribosome biogenesis was originally thought to merely reflect the increased growth and proliferation rates in cancer cells, today it is well accepted that dysregulation in ribosome biogenesis is a result of increased activity of oncogenes or inactivated tumor suppressors [28]. One of the best understood cellular surveillance mechanisms in this context is the nucleolar stress response, also referred to as the ribosomal surveillance pathway, that often results in activation of p53 [28]. The tumor suppressor p53 restrains tumor growth by inducing cell cycle arrest, senescence, or apoptosis [159]. Therefore, it is not surprising that p53 is mutated or functionally inactivated in most human cancers [160]. Targeting p53 by activating wildtype p53 or restoring the activity of loss-of-function mutants has become a promising strategy in cancer treatment. Classical chemotherapeutics, such as alkylating agents or platinum-based drugs, in general target cancer cells by directly or indirectly inducing DNA damage in rapidly proliferating cells, which leads to activation of p53 [161]. Interestingly, 21 of 36 screened chemotherapeutics, including doxorubicin and camptothecin, were shown to additionally inhibit various steps of ribosome biogenesis, leading to p53 stabilization [162]. Since classical chemotherapeutics discriminate between cancer cells and healthy cells by their different proliferative index, patients frequently suffer from side effects on other proliferating cells in the body, which includes bone marrow suppression, alopecia, mucositis, as well as toxicity to the gastrointestinal tract, skin, and heart [163]. In addition, long-term adverse effects, such as infertility or development of secondary cancers were observed in patients following chemotherapy [164]. The finding that p53 can also be activated in a non-genotoxic manner by disrupting nucleolar function and the aim to overcome adverse effects, gave rise to the development of a series of small molecule inhibitors, which selectively inhibit RNA Pol I transcription [165,166]. One of the most promising of such inhibitors is CX-5461, which impedes the selectivity factor SL1 from binding to the rDNA promoter, thereby preventing recruitment of the Pol I complex to the rDNA and initiation of transcription [167]. Apart from this, CX-5461 stabilizes G-quadruplex structures with increased toxicity in *BRCA*-deficient or PARP inhibition resistant cancer cells [168]. In vitro studies have revealed a high antiproliferative effect of CX-5461 in a wide range of human cancers, with those derived from p53 wildtype hematological malignancies being the most sensitive [167]. By using the Eμ-*Myc* mouse model of Burkitt’s lymphoma, which is an aggressive type of lymphoma affecting B lymphocytes, it was shown in vivo that treatment with CX-5461 has the potential to lead to an almost complete disease remission and a significantly increased survival [165]. Importantly, the normal B cell population in those mice was maintained, suggesting that CX-5461 selectively targets cancer cells. Due to the promising results of this preclinical data, CX-5461 is currently tested in several clinical trials. A phase I clinical trial has successfully been completed in patients with hematological cancers and at present phase I/II trails are undergoing for solid tumors, including metastatic breast cancer, ovarian, and pancreas cancer [169]. Thus, selectively targeting ribosome biogenesis could provide a novel and efficient strategy in cancer therapy. Most cancer types rely on increased levels of ribosomes and therefore display higher sensitivity towards inhibition of ribosome biogenesis compared to normal cells. Thus, in contrast to existing chemotherapies, inhibition of ribosome biogenesis is likely less genotoxic to the non-tumor population of cells, which is associated with a reduced risk of adverse effects. The previously mentioned findings from studies with CX-5461 strongly support this notion. Given the high complexity of ribosome biogenesis and the variety of factors, which are involved in this process, it is likely that in future even more effective small molecule inhibitors will be identified.

As described in Section 3, PARP inhibitors have successfully entered the clinic as monotherapeutic agents, as well as in combination with cytostatic chemotherapy or radiotherapy. PARP inhibitors can act by inducing synthetic lethality in cancers that are deficient in HR-mediated DNA repair, e.g., loss-of-function mutation in *BRCA* [128,129]. More recent studies have suggested that PARP inhibitors may also induce replication stress and subsequent DNA damage [95,170,171]. Development of chemo-resistance has been proven to be a major problem in the clinical efficacy of PARP inhibitors [172]. Extensive in vitro and in vivo studies have identified several potential resistance mechanisms, including reactivation of HR, upregulation of drug efflux pumps and stabilization of replication forks [131].

Interestingly, some recent studies suggested that targeting nucleolar proteins/processes in combination with PARP inhibitor treatment may be beneficial for the treatment of some cancers, e.g., by overcoming PARP inhibitor resistance mechanisms. Thus, in a recent study CX-5461 was demonstrated to induce replication stress and activate the DNA damage response in high-grade serous ovarian cancer (HGSOC) cells [173]. CX-5461 showed significant therapeutic benefit as a single agent in HGSOC-patient-derived xenografts with reduced sensitivity to PARP inhibitors by overcoming replication fork protection, which is a well-known PARP inhibitor resistance mechanism. Importantly, the combination of CX-5461 and PARP inhibitors resulted in enhanced replication stress, DNA damage and cell death and exhibited great therapeutic efficacy, especially in HR-deficient HGSOC-patient-derived xenografts. Thus, there is evidence that combining PARP inhibitors with CX-5461 could improve treatment of HR-deficient HGSOC.

Furthermore, a novel mechanism for PARP resistance development has been reported by Sun et al. [174], which involves increased phosphorylation of the ribosomal protein S6 (RPS6). RPS6 is a component of the 40S ribosomal subunit and a well-known downstream effector of the mammalian target of rapamycin (mTOR) signaling pathway, which is the major nutrient-sensitive regulator of eukaryotic cell growth, metabolism, proliferation and survival [175]. RPS6 is phosphorylated by ribosomal protein S6 kinases (S6Ks) at five C-terminal serine sites and this modification is crucial for regulation of cell size, cell proliferation and glucose homeostasis [176]. Phosphorylation of RPS6 is greatly increased in *BRCA1*-deficient cancer cells, which are resistant to PARP inhibition [174]. Importantly, in *BRCA1*-deficient cells RPS6 phosphorylation promoted loading of the HD marker RAD51 onto DNA following IR-induced DNA damage. Thus, RPS6 phosphorylation might play a key role in PARP inhibitor resistance by regulating HR. Intriguingly, rapamycin, which is a clinically used selective inhibitor of mTOR and S6 phosphorylation, could restore sensitivity towards PARP inhibition, suggesting that combined inhibition of S6 phosphorylation and PARP could be efficient in cancers with PARP inhibitor resistance and HR defects, including *BRCA1*-deficient breast and ovarian cancers.

Several studies have reported that *BRCA* mutations or other HR-mediated DNA repair deficiencies are not mandatory for the clinical effectiveness of PARP inhibitors in cancer therapy [177,178,179]. In accordance with this, Kim et al. [112] revealed an alternative working mechanism of PARP inhibitors, which is independent of HR, DNA damage and replication stress. Mechanistically, it was shown that snoRNAs can stimulate PARP1 enzymatic activity in the nucleolus, resulting in ADP-ribosylation of DDX21. The DEAD-box RNA helicase DDX21 was previously reported to directly interact with rRNA and snoRNAs at the transcribed rDNA locus, thereby promoting rRNA synthesis, rRNA processing and modification [180]. Furthermore, analyses of gene expression profiles from breast cancer patients identified DDX21 as a prognostic marker in breast cancer [181]. Kim et al. [112] demonstrated that DDX21 promotes rDNA transcription and breast cancer growth upon PARP1-mediated ADP-ribosylation. PARP inhibition on the other hand resulted in reduced tumor cell growth by modulating rRNA levels, DDX21 ADP-ribosylation and DDX21 localization. These findings could be explained by the fact that some cancer types are “addicted” to ribosome biogenesis, whereas reducing ribosome biogenesis can counteract cancer cell growth. The finding that PARP inhibitors can reduce tumor growth by targeting ribosome biogenesis provides a mechanistic explanation for efficacy of PARP inhibitors in cancer cells lacking deficiencies in HR. In addition, these results further suggest that DDX21 nucleolar localization could be a predictive biomarker of clinical responses to PARP inhibition.

## 6. Concluding Remarks and Perspectives

Nucleolar localization of PARP1 was demonstrated almost four decades ago. For many years, scientists have been puzzled whether PARP1 accumulation in the nucleolus merely occurs for storage reasons or if PARP1 might also be implicated in nucleolar functions. As summarized in this review, there is growing evidence, supporting a role of PARP1 and PARylation in nucleolar biology. PARP1 is implicated in multiple areas of nucleolar function, including maintenance of nucleolar integrity and structure, regulation of Pol I transcription, establishment of silent rDNA chromatin, as well as regulation of DNA damage-induced nucleolar-nucleoplasmic shuttling processes of key genome maintenance factors, e.g., WRN and XRCC1 (Figure 1). Previous studies have demonstrated that PAR can function as a seed of liquid-liquid demixing processes, thereby triggering the formation of biomolecular condensates [124,125]. Therefore, it can be assumed that PARP1 and PARylation might regulate the general biophysical state of nucleolar structure. Since PARP1 plays a crucial role in several DNA repair pathways, of which at least NHEJ and HR have been reported to take place in the nucleolus, it can be anticipated that PARP1 might also be involved in the repair of rDNA [88,182]. Repetitive rDNA sequences represent one of the most unstable regions in the genome [6]. Instability of rDNA is associated with severe pathological conditions, including cancer, premature aging and neurological impairments [182]. Yet, the DNA damage repair mechanisms that govern genomic stability and maintenance in the nucleolus remain elusive. In future studies, recent advancements in CRISPR-genome engineering could provide deeper insights into the mechanisms that help cells keep their rDNA intact. Until now, in large, only PARP1 was reported to play a role in nucleolar biology. In future studies it will be interesting to investigate to what extent other members of the PARP family, e.g., PARP2, also contribute to nucleolar functions. As previously described, the tripartite organization of the nucleolus reflects the different stages of ribosome biogenesis. Therefore, identifying the exact localization of PARP1 and other PARPs, as well as PAR, in nucleolar substructures could provide further evidence for a role in certain steps of ribosome biogenesis. Importantly, PARP1 inhibitors have entered the clinic as promising chemotherapeutic agents in the treatment of various cancer types, mainly by exploiting synthetic lethal interactions between PARP inhibition and defects in genes that are responsible for HR-mediated DNA repair. As discussed above alternative working mechanisms of PARP inhibitors are conceivable, which act by preventing tumor growth through inhibition of ribosome biogenesis [112]. These data indicate that PARP inhibitors might be effective in a broader spectrum of cancer types than originally anticipated. In addition, in this study, the DEAD-box RNA helicase DDX21 was identified as a potential marker to predict sensitivity towards PARP inhibitors, which needs to be further evaluated in clinical trials. In future studies it will be important to elucidate further mechanisms by which PARP inhibitors contribute to inhibition of nucleolar functions, to pave the way for the identification of other biomarkers, which can predict response to PARP inhibitors in cancer patients.
